# Pain Control: Normalization of the BPCQ Questionnaire on a Group of Patients Diagnosed with Malignant Cancer

**DOI:** 10.3390/ijerph182413069

**Published:** 2021-12-11

**Authors:** Aleksandra Czerw, Urszula Religioni, Filip Szymański, Katarzyna Sygit, Krzysztof Zdziarski, Dominika Mękal, Grażyna Dykowska, Anna Kłak, Agnieszka Barańska, Piotr Merks, Mariola Borowska, Elżbieta Cipora, Monika Pajewska

**Affiliations:** 1 Department of Health Economics and Medical Law, Medical University of Warsaw, 02-091 Warsaw, Poland; grazyna.dykowska@wum.edu.pl (G.D.); mariola.borowska@wum.edu.pl (M.B.); 2 Department of Economic and System Analyses, National Institute of Public Health NIH—National Research Institute, 00-791 Warsaw, Poland; mpajewska@pzh.gov.pl; 3 Collegium of Business Administration, Warsaw School of Economics, 02-513 Warsaw, Poland; urszula.religioni@gmail.com; 4 Department of Civilization Diseases, Faculty of Medicine, Collegium Medicum, Cardinal Stefan Wyszynski University in Warsaw, 01-938 Warsaw, Poland; f.szymanski@uksw.edu.pl; 5 Faculty of Health Sciences, Calisia University, 62-800 Kalisz, Poland; ksygit@poczta.onet.pl; 6 Department of Social Medicine and Public Health, Pomeranian Medical University in Szczecin, 70-204 Szczecin, Poland; krzysztof.zdziarski@pum.edu.pl; 7 Department of Oncological Prevention, Medical University of Warsaw, 02-091 Warsaw, Poland; dmekal@wum.edu.pl; 8 Department of Environmental Hazards Prevention, Allergology and Immunology, Medical University of Warsaw, 02-091 Warsaw, Poland; klak.anna2@gmail.com; 9 Department of Computer Science and Medical Statistics, Medical University of Lublin, 20-090 Lublin, Poland; agnieszkabaranska@umlub.pl; 10 Faculty of Medicine, Collegium Medicum, Cardinal Stefan Wyszynski University in Warsaw, 01-938 Warsaw, Poland; piotrmerks@googlemail.com; 11 Faculty of Medical Science, Jan Grodek State University in Sanok, 38-500 Sanok, Poland; elacipora@interia.pl

**Keywords:** pain, pain control, BPCQ assessment, normalization, cancer

## Abstract

The purpose of this article is to examine the applicability of the Beliefs about Pain Control Questionnaire (BPCQ) among cancer patients and develop norms that allow differentiation of patients with diagnosed cancer in terms of beliefs about pain control. Normalization aims to establish the value of test results in the study population. The study involved 1187 patients diagnosed with cancer in outpatient care Maria Sklodowska-Curie Cancer Center and Institute of Oncology, in Warsaw. The applied tool was the Beliefs about Pain Control Questionnaire developed by S. Skevington. The results are most strongly differentiated in each dimension of pain control by education, income, and professional status. Sten norms were developed to determine the level of beliefs about pain control in low, average, and high categories. The BPCQ assessment applies to cancer patients, and the assessment of the location of pain control in patients will allow for the identification of patients whose standard therapy should be supplemented with psychotherapeutic support.

## 1. Introduction

Cancer is the second cause of death in Poland, and forecasts indicate that in the next decade, cancer will be the leading cause of death [1,2]. Additionally, 18.1 million new incidents of cancer and 9.6 million deaths were registered worldwide in 2018 [3].

Cancer is associated with various ailments, the most common of which is pain. According to the definition of the International Association for the Study of Pain (IASP), pain is an unpleasant sensory and emotional experience associated with current or potential tissue damage or described in terms of such damage.

Studies show that 30–50% of people experience pain during active cancer therapy, and even up to 90% of patients in the later stages of the disease. Moderate-to-severe pain occurs in approximately 40% of patients in moderate stages of the disease, and pain attacks in approximately 80% of patients in advanced stages of cancer [4].

Pain has its cognitive, emotional, and behavioral consequences. Pain is a subjective and multidimensional experience, which is why there are significant difficulties in its direct and objective assessment [5]. The pain response is not innate and automatic. It is a result of the patient’s beliefs and reaction to pain and, as studies show, it can be significantly modified by the social environment [4]. Psychological factors play a crucial role in reaction to pain [6]. There are studies that explored the relationship between hope and pain among oncology patients; however, it is important to take into account biological, psychological, and social contexts as well [7,8].

Among other factors, the localization of sense of control is also a variable associated with pain [9]. The sense of control is an individual human property that can be treated as a personality trait that can be represented on a continuum from a sense of external control to a sense of internal control. Persons with an internal location of control (inner directed) recognize that they exercise control, are responsible, and treat encountered situations as the result of their own actions. Persons with an external location of control (other directed) feel less personal responsibility and more often grant it to external factors, i.e., happiness, chance, or actions of others [5].

Studies indicate the relationship between the location of health control and undertaking health behaviors [9,10]. The internal location of control, i.e., convincing the patient about his/her own impact on the course of the illness, increases the sense of agency and thus promotes undertaking pro-health behaviors and taking responsibility for their own health [11], although it is indicated that people with an internal location of health control are more responsible for their health, but only when they treat it as a value [12].

Beliefs and the attitude of patients toward pain affect the severity of pain, as well as the feeling of other symptoms associated with the illness, compliance with medical recommendations, and the final results of treatment [13,14,15]. Studies also emphasize the impact of the internal pain control factor on patient optimism and the perception of positives in their illness [16]. In turn, the external location of control affects the search for medical information, but it is not associated with the desire to independently solve health problems and the tendency to accept the possibility of active participation in treatment [10].

Beliefs about pain control are measured using the Beliefs about Pain Control Questionnaire (BPCQ), developed by S. Skevington [11]. The questionnaire is used in the examination of adults complaining of pain, both at the stage of diagnosis and therapy. It measures the strength of individual beliefs about controlling pain personally (internal factors), through the influence of physicians (the strength of others), and through random events.

The main objective of the study is the normalization of the Beliefs about Pain Control Questionnaire (BPCQ) for cancer patients. Normalization allows norms regarding test results to be established for a specific population. This procedure allows for an understanding of what the result obtained by a specific person in the questionnaire means, including whether the result is high or low.

## 2. Materials and Methods

### 2.1. Characteristics of the Studied Normalization Group

The normalization sample included results of 1187 respondents aged 21–96 (M = 62.12; SD = 14.03), 666 women aged 21–96 (M = 58.17; SD = 12.88) and 521 men aged 22–96 (M = 67.12; SD = 13.75). The criteria for enrolling patients were the patient’s availability at the Maria Sklodowska-Curie Cancer Center and Institute of Oncology, in Warsaw, for the duration of the study and the patient’s consent to participate in the study.

The structure of diagnoses among the studied women was as follows: breast cancer, 29%; ovarian cancer, 25.8%; endometrial cancer, 17.4%; colorectal cancer, 14.9%; stomach cancer, 6.8%; pancreatic cancer, 3.6%; bladder cancer, 2.6%. In the studied group of men, the following types of cancer occurred: prostate cancer, 43.8%; colorectal cancer, 26.9%; bladder cancer, 15.9%; stomach cancer, 9.2%; pancreatic cancer, 4.2% (Table 1).

In the studied group, the majority of patients had secondary education (37.7%) or higher education (33.1%). The largest group of people lived in cities over 500,000 inhabitants (36.0%), and the average monthly income per person in a household was over 2000 PLN for 27.7% of people, 1001–1500 PLN for 26.7% of people, and 1501–2000 PLN for 25.2% of people. The majority of patients were pensioners (52.1%), but a large number of participants were also professionally active (39.1%). Patients were mainly married (68.4%). Detailed information about the sample is presented in Table 2.

### 2.2. Tool Applied

The BPCQ assessment, measuring the strength of individual beliefs about pain control, consists of 13 statements that make up three factors of pain control:

Internal factors (a belief in pain control personally);

Influence of physicians (a belief that pain is controlled by others);

Random events (a belief that there is no self-influence on pain control).

The questionnaires used in the study are the only version of the BPCQ test available in Poland through the Psychological Testing Laboratory of the Polish Society of Psychology (translation by Juczyński) [17]. The statements in the questionnaire should be assessed by the patient at a six-point Likert scale, where 1 means “no, I completely do not agree” and 6 indicates “yes, I totally agree”. The results of the questionnaire are not given in the form of one indicator, but for each dimension of the location of control, the sum of the results is calculated separately. The range of points possible to obtain is from 5 to 30, in the area of external control, and from 4 to 24 in the areas of influence of physicians and random events. The higher the score of a given area, the stronger the belief that pain can be controlled by the patient through the strength of a given factor.

The Cronbach’s alpha internal compatibility index, according to Juczyński, was 0.75 for the whole scale and 0.86 for the influence of physicians, 0.82 for the area related to internal factors, and a low value of 0.58 for the impact of random events [17]. In the current sample, Cronbach’s alpha coefficients were equal to 0.81 for the whole scale and 0.78 for the influence of physicians, 0.82 for the area related to internal factors, and 0.66 for the impact of random events.

Statistical analysis was performed with the use of IBM SPSS 25.0 software. The analysis regarding between groups comparisons was based on the values of an independent *t*-test when comparing two groups (gender, city/town size, working people vs. retirees/pensioners) and on the values of one-way analysis of variance when analyzing education. The analysis of variance was followed by Gabriel’s post hoc test which was designed for analyzing unequal groups [18]. The groups of participants with primary and vocational education were combined in order to avoid analyzing very small groups (participants with primary education). Students, homemakers, and unemployed patients were excluded from the analysis concerning professional status due to the small number of participants in these three groups. Correlations were analyzed with the use of Pearson’s correlation coefficient.

## 3. Results

Table 3, Table 4 and Table 5 present descriptive statistics for the intensification of the internal location of pain control (Table 3), the location of pain control in the influence of physicians (Table 4), and the location of pain control in random events (Table 5) depending on demographic variables along with the values of tests concerning the significance of statistical differences.

Based on Gabriel’s post hoc test, it was found that statistically significant differences in the internal location of pain control occurred between the group of people with primary or vocational education (M = 16.38) and the group of people with higher education (M = 14.12) (*p* < 0.05). It was found that the intensity of the location of pain control in the influence of physicians was the lowest in the group of people with higher education (M = 15.50), compared with the average of people with secondary education (M = 16.77) and primary/vocational (M = 17.04); it was lower in the group of people with a monthly net income per person in the family above 1500 PLN (M = 16.00), compared with people with an income of up to 1500 PLN (M = 16.90) and lower in the group of working people (M = 15.74) than retirees/pensioners (M = 17.03).

It was found that the intensity of the location of pain control in random events was the lowest in the group of people with higher education (M = 14.12) in comparison with people with secondary education (M = 15.66) and primary/vocational education (M = 16.38). The location of pain control in random events was also lower in the group of people with a monthly net income per person in the family above 1500 PLN (M = 14.69), compared with people with lower income (M = 16.09), and lower in the group of employed people (M = 14.71) than in the group of retired people/pensioners (M = 15.97).

No statistically significant differences between the genders were found; therefore, the norms were determined for the whole sample studied together. Based on the value of the Pearson correlation coefficient r, no statistically significant correlation was found between the age of the respondents and the internal location of pain control, r (548) = 0.08, *p* > 0.05. Correlation between the age of the respondents and the location of pain control for the group attributing control to the influence of physicians was statistically significant but weak. Identically, age and the location of pain control for the group attributing control to random events, was statistically significant and positive but weak. It was, respectively, r (548) = 0.15, *p* < 0.001 for the location of pain control in the group attributing control to the influence of physicians and r (548) = 0.17, *p* < 0.001 for the location of pain control in the group attributing control to random events.

Analysis of the results did not show statistically significant differences in the method of pain control depending on the type of patient treatment or metastases (in all cases *p* < 0.05).

### Norms

Table 6, Table 7 and Table 8 present values of sten and centile norms determined in the studied sample using the calculated weight for the results on the scale of internal control location (Table 6), the location of control in the group attributing control to the influence of physicians (Table 7), and those attributing control to random events (Table 8). Sten scores from 1 to 3 sten should be interpreted as low, from 4 to 7 sten as average, and from 8 to 10 sten as high.

## 4. Discussion

Each disease is associated with some discomfort and limitations, and many diseases are accompanied by pain [19]. Cancer is particularly associated with the experience of pain.

Numerous studies show that psychosocial factors, socio-cultural, cognitive, and emotional–motivational processes play a significant role in experiencing pain. Great importance is attributed to the location of pain control. In psychology, the sense of being under control indicates the patient’s belief that he or she has an influence on a number of events in life, including achieving his/her goals [20,21].

Results of the authors’ study show that individual dimensions of pain control are strongly conditioned by the level of education, professional status, and income of patients. A high inner sense of control is associated with better mental well-being, which is observed, among others, in chronically ill patients, which is emphasized by a number of authors, including Wallston and Thompson [10,21]. This information is a valuable source, especially for clinical psychologists, who can more easily identify the groups of people who should receive psychological support, especially in the area of increasing self-confidence in the fight against the disease.

People with an internal location of control are more prone to broadening their knowledge about health [22], active ways of coping, as well as health-related behaviors such as taking medications regularly, regular follow-ups, following a diet, or giving up smoking [12]. It is believed that this approach reduces hopelessness and depressive symptoms and promotes greater activity in an individual and helps them better cope with pain [23,24].

In turn, people with a low sense of internal control more often negate symptoms of the disease and the need to comply with health-promoting behaviors. These people may trust others (influence of physicians) or believe that all events are random [10]. An external location of health control is generally associated with chronic negative emotions experienced by patients: depression, hostility, anxiety, as well as other more strongly felt symptoms associated with the disease. People with an external location of control use passive ways of coping with pain and illness and are less likely to choose positive forms of adaptation to the illness [10,19]. People with an external location of control usually do not care about expanding knowledge about their health [10,22,23].

Juczyński also indicates a link between the location of pain control and acceptance of the disease—patients who accept the disease less often attribute pain to the influence of physicians than patients who do not accept the disease [17].

The results of the authors’ study indicate that patients diagnosed with chronic pain, migraine, multiple sclerosis, and malignant neoplasms obtained average results (close to the center of a scale used) in each area of pain control. Some studies prove that the external location of control is more common among women than men [19,25], although such a relationship was not found in the authors’ study, or the study conducted by Basińska et al. [12].

In the study conducted by Basińska et al., among patients with lung cancer, the dominant location of control concerned the area of influence of physicians (M = 17.08; SD = 4.97), which was also observed among patients with colorectal cancer (M = 16.98; SD = 4.32). In both groups, patients attributed the least importance to the impact of random events [12]. In the authors’ study, patients also obtained slightly higher results for the areas of the impact of physicians and internal factors than for the random events.

In the study of cancer patients, both treated causally as well as symptomatically, it was observed that patients treated symptomatically placed control in the area of the influence of physicians (M = 20.23; SD = 3.44) and random events (M = 17.90; SD = 3.93) in comparison with causally treated patients (respectively, M = 17.80 and SD = 2.89 for the impact of physicians; M = 15.05 and SD = 3.08 for random events). In the same study, it was demonstrated that patients at the terminal stage assigned much more importance to physicians and random events than to internal factors. It was also noted that people dominated by anxiety attributed a greater role to the influence of physicians or random events, while people showing a positive approach to the disease were more often characterized by an internal location of pain control [4].

BPCQ test results were varied among other groups of patients. Patients with chronic lower limb ischemia attributed the greatest importance in pain control to the influence of physicians (M = 19.04). Patients attributed less importance to random events. The average point value described the impact of random events (M = 16.85), and these beliefs were reflected by a significant share of lifestyle risk factors. Patients who were not convinced of their own influence on the control of ischemic pain did not take responsibility for their own health and well-being. They undertook behaviors unfavorable for the course of the disease, including smoking even though it increased the level of pain [26].

Additionally, among people with spine diseases, the external location of control dominated (mainly in the area of random events) [22]. In another study involving patients in chronic pain outpatient clinics (mainly cancer patients), it was found that the location of pain control in patients was affected by sociodemographic factors such as age, education, and professional status. Among persons under 40 years of age, patients controlled pain mainly through internal factors, but among older patients, the influence of physicians dominated. Patients’ beliefs about self-control as a method of controlling pain increased with the degree of education. In turn, random events were of the least importance among people with higher education. For employed people and retirees, physicians constituted the most important factor in pain control [27,28].

In patients with osteoarthritis of the spine, it was also noted that people with higher education less often pointed to the significant influence of physicians on the perception of pain. A significant relationship was also observed in relation to the gender of the respondents—men more often indicated the role of internal pain control [23].

Among patients after cardiac surgery, older patients (M = 17.1; SD = 2.21), compared with younger patients (M = 10.5; SD = 3.53), placed greater emphasis on the influence of physicians in controlling pain, and they also attributed a greater role to random events, which in older people was at the level of M = 16.1 (SD = 2.9), while in younger people, M = 12.0 (SD = 2.82) [29,30].

In turn, a study involving patients with a history of chronic pain lasting over 12 weeks conducted at a pain clinic indicates that patients’ behavior, related to the sensation of pain, as well as the results of treatment, is influenced by their knowledge about health (health literacy), including the ability to understand it and implement specific health-promoting behaviors [31]. This study indicates that patients during the treatment process need understandable knowledge about their clinical condition and justification of decisions made by medical staff regarding the selection of specific therapies, which can significantly affect faith in their own strength and an increase in the internal dimension of pain control. Changing attitudes and beliefs of patients regarding pain are considered to be an improvement in the treatment of chronic pain. Multidisciplinary approaches to pain, taking into account psychological aspects by changing the approach to one’s illness and perceived pain, allow for the reduction in the perception of pain and improvement of treatment effectiveness [32].

### Limitations

The authors’ study has limitations; firstly, the influence of the stage of the disease or the type of treatment was not taken into account in the results obtained. Further research in this area may reveal additional links between pain control and other factors that affect the type of perception of pain control in a patient.

## 5. Conclusions

Studies on pain should take into account the biological, psychological, and social contexts. Pain is a psychosomatic symptom that requires both the use of appropriate medications and, above all, psychological support. The perception of pain by patients is not homogeneous, and therefore, patients with the same disease should not be treated equally in terms of their approach to pain relief.

The tool used in the assessment of pain control of cancer patients is the Beliefs about Pain Control Questionnaire. The assessment of the location of pain control translates into the level of pain experienced by patients, assessment of patients’ coping resources, or the effectiveness of using these resources in the treatment process. This kind of approach to patients allows for the isolation of the group of patients who will require psychological support as a supplement to standard treatment.

The conducted study allowed the norms (mean results) of the BPCQ assessment o be established for oncological patients. This information is especially valuable for psychologists working with patients. Knowledge of norms and sociodemographic variables that may affect the results allows health professionals to distinguish those groups of patients who have, for example, a low sense of self-control of health and thus should receive psychological support.

## Figures and Tables

**Table 1:** Types of cancer in the studied group and in the population.

	Women			Men		
Cancer Location	Population (%)	Sample (%)	Weight	Population (%)	Sample (%)	Weight
Breast cancer	21.9	29.0	0.76	0	0	-
Ovarian cancer	4.7	25.8	0.18	0	0	-
Stomach cancer	2.4	6.8	0.35	4.5	9.2	0.49
Colorectal cancer	10.1	14.9	0.68	12.2	26.9	0.45
Prostate cancer	0	0	-	15.5	43.8	0.35
Bladder cancer	2.0	2.6	0.77	6.9	15.9	0.43
Endometrial cancer	7.3	17.4	0.42	0	0	-
Pancreatic cancer	2.2	3.6	0.61	2.3	4.2	0.55

**Table 2:** Characteristics of the studied normalization sample.

Characteristic	*n*	%
Education		
Primary	97	8.3
Vocational	250	21.1
Secondary	447	37.7
Higher	393	33.1
Place of residence		
Village	221	18.6
City up to 20,000 inhabitants	131	11.0
City up to 50,000 inhabitants	154	13.0
City up to 100,000 inhabitants	144	12.1
City up to 500,000 inhabitants	110	9.3
City above 500,000 inhabitants	427	36.0
Average monthly income		
No data	8	0.7
Below 500 PLN	24	2.0
501–1000 PLN	210	17.7
1001–1500 PLN	317	26.7
1501–2000 PLN	299	25.2
Above 2000 PLN	329	27.7
Professional status		
Working	464	39.1
Student	19	1.6
Retired/pensioner	618	52.1
Homemaker	53	4.5
Unemployed	33	2.8
Marital status		
Unmarried	93	7.8
Married	812	68.4
Widow/widower	192	16.2
Divorced	90	7.6
Total	1187	100

**Table 3:** Descriptive statistics for the intensification of the internal location of control depending on demographic variables.

		M	SD	Min	Max	Test	*p*
Gender	Women	16.11	5.39	5	30	*t* (550) = −0.82	0.410
	Men	16.51	5.68	5	30		
Education	Prim./voc.	17.08	5.73	5	30	F (2.549) = 3.15	0.044
	Secondary	16.25	5.55	5	30		
	Higher	15.58	5.16	5	30		
City/town	Up to 100.000 inhabitants	16.27	5.52	5	30	*t* (550) = 0.04	0.972
size	Above 100.000 inhabitants	16.26	5.49	5	30		
Net	Up to 1.500 PLN	16.55	5.45	5	30	*t* (550) = 1.18	0.239
income	Above 1.500 PLN	16.00	5.54	5	30		
Professional	Working	16.12	5.09	5	30	*t* (502) = −0.67	0.501
status	Student	17.57	7.25	8	30		
	Retired/pensioner	16.45	5.73	5	30		
	Homemaker	15.71	5.18	6	28		
	Unemployed	15.21	6.86	5	26		
	Total	16.27	5.50	5	30		

M—median; SD—standard deviation; min—minimum value; max—maximum value; *t*—Student’s *t*-test value for independent samples; F—one-way analysis of variance value; *p*—statistical significance.

**Table 4:** Descriptive statistics for the intensification of the location of pain control in the influence of physicians depending on demographic variables.

		M	SD	Min	Max	Test	*p*
Gender	Women	16.42	4.58	4	24	*t* (550) = −0.07	0.943
	Men	16.45	4.84	4	24		
Education	Prim./voc.	17.04	4.98	4	24	F (2.549) = 5.52	0.004
	Secondary	16.77	4.37	4	24		
	Higher	15.50	4.65	4	24		
City/town	Up to 100.000 inhabitants	16.45	4.64	4	24	*t* (550) = 0.07	0.947
size	Above 100.000 inhabitants	16.42	4.73	4	24		
Net	Up to 1.500 PLN	16.90	4.67	4	24	*t* (550) = 2.29	0.023
income	Above 1.500 PLN	16.00	4.65	4	24		
Professional	Working	15.74	4.40	4	24	*t* (502) = −3.12	0.002
status	Student	16.75	5.34	8	24		
	Retired/pensioner	17.03	4.73	4	24		
	Homemaker	15.41	5.66	5	24		
	Unemployed	16.68	4.29	9	24		
	Total	16.43	4.68	4	24		

M—median; SD—standard deviation; min—minimum value; max—maximum value; *t*—Student’s *t*-test value for independent samples; F—one-way analysis of variance value; *p*—statistical significance.

**Table 5:** Descriptive statistics for the intensification of the location of pain control in random events depending on demographic variables.

		M	SD	Min	Max	Test	*p*
Gender	Women	15.47	4.48	4	24	*t* (550) = 0.62	0.534
	Men	15.22	4.83	4	24		
Education	Prim./voc.	16.38	4.62	4	24	F (2.549) = 11.12	0.001
	Secondary	15.66	4.36	4	24		
	Higher	14.12	4.68	4	24		
City/town	Up to 100.000 inhabitants	15.52	4.58	4	24	*t* (550) = 0.85	0.395
size	Above 100.000 inhabitants	15.18	4.68	4	24		
Net	Up to 1.500 PLN	16.09	4.47	4	24	*t* (550) = 3.61	0.001
income	Above 1.500 PLN	14.69	4.66	4	24		
Professional	Working	14.71	4.53	4	24	*t* (502) = −3.07	0.002
status	Student	15.56	6.09	6	24		
	Retired/pensioner	15.97	4.60	4	24		
	Homemaker	15.00	4.32	5	23		
	Unemployed	13.89	5.06	4	24		
	Total	15.37	4.62	4	24		

M—median; SD—standard deviation; min—minimum value; max—maximum value; *t*—Student’s *t*-test value for independent samples; F—one-way analysis of variance value; *p*—statistical significance.

**Table 6:** Raw results and corresponding results normalized for the results on the scale internal location of control.

Results	Value	Sten	Centile
Low	5	1	1
	6	2	4
	7	2	6
	8	3	8
	9	3	10
	10	3	14
Average	11	4	18
	12	4	22
	13	4	28
	14	5	34
	15	5	41
	16	5	49
	17	6	55
	18	6	62
	19	6	69
	20	7	75
	21	7	81
High	22	8	85
	23	8	88
	24	8	91
	25	9	94
	26	9	96
	27	9	98
	28	10	99
	29	10	99
	30	10	100

**Table 7:** Raw results and corresponding results normalized for the results on the scale of location of pain control in the group attributing control to the influence of physicians.

Results	Value	Sten	Centile
Low	4	1	1
	5	1	2
	6	2	2
	7	2	3
	8	2	4
	9	2	6
	10	3	9
	11	3	13
Average	12	4	17
	13	4	22
	14	4	29
	15	5	37
	16	5	46
	17	6	55
	18	6	62
	19	6	68
	20	7	76
	21	7	82
High	22	8	86
	23	8	90
	24	9	96

**Table 8:** Raw results and corresponding results normalized for the results on the scale of location of pain control in the group attributing control to random events.

Results	Value	Sten	Centile
Low	4	1	1
	5	2	2
	6	2	3
	7	2	5
	8	3	7
	9	3	10
	10	3	13
Average	11	4	17
	12	4	23
	13	4	29
	14	5	37
	15	5	45
	16	6	53
	17	6	62
	18	6	69
	19	7	77
	20	7	84
High	21	8	89
	22	8	93
	23	9	96
	24	10	98
